# High urban NO*_x_* triggers a substantial chemical downward flux of ozone

**DOI:** 10.1126/sciadv.add2365

**Published:** 2023-01-18

**Authors:** Thomas Karl, Christian Lamprecht, Martin Graus, Alexander Cede, Martin Tiefengraber, Jordi Vila-Guerau de Arellano, David Gurarie, Donald Lenschow

**Affiliations:** ^1^ Department of Atmospheric and Cryospheric Sciences, University of Innsbruck, Innsbruck, Austria.; ^2^ Luftblick, Innsbruck, Austria.; ^3^ Meteorology and Air Quality Section, Wageningen University, Wageningen, Netherlands.; ^4^ Case Western Reserve University, Cleveland, OH, USA.; ^5^ National Center for Atmospheric Research, Boulder, CO, USA.

## Abstract

Nitrogen oxides (NO*_x_*) play a central role in catalyzing tropospheric ozone formation. Nitrogen dioxide (NO_2_) has recently reemerged as a key target for air pollution control measures, and observational evidence points toward a limited understanding of ozone in high-NO*_x_* environments. A complete understanding of the mechanisms controlling the rapid atmospheric cycling between ozone (O_3_)–nitric oxide (NO)–NO_2_ in high-NO*_x_* regimes at the surface is therefore paramount but remains challenging because of competing dynamical and chemical effects. Here, we present long-term eddy covariance measurements of O_3_, NO, and NO_2_, over an urban area, that allow disentangling important physical and chemical processes. When generalized, our findings suggest that the depositional O_3_ flux near the surface in urban environments is negligible compared to the flux caused by chemical conversion of O_3_. This leads to an underestimation of the Leighton ratio and is a key process for modulating urban NO_2_ mixing ratios. As a consequence, primary NO_2_ emissions have been significantly overestimated.

## Teaser

Ozone deposition is negligible due to the chemical transformation of ozone controlled by atmospheric turbulence in high NOx.

## INTRODUCTION

Nitrogen oxides (NO and NO_2_) play a crucial role for controlling the oxidizing power of the atmosphere (*1*, *2*). More recently, space-based remote-sensing observations of NO_2_ have become a tractable tool to monitor the catalytic efficiency of the atmosphere (*3*, *4*). The complex chemical cycling, however, still presents a challenge for accurate ozone predictions in polluted environments (*5*, *6*). Nitrogen oxides have also emerged as a primary public health concern in many regions (*7*, *8*), and significant efforts have been underway to understand the fate and chemical cycling between O_3_-NO-NO_2_ in urban areas (*9*, *10*–*12*). Because NO_2_ is typically identified as an irritant of the respiratory tract (*13*), atmospheric levels are regulated and subject to a significant policy debate (*14*, *15*). World Health Organization (WHO) air quality (AQ) standards have been implemented in Europe as regulatory action under the EU Thematic Strategy on Air Pollution, which aims to limit urban street canyon NO_2_ concentrations to 40 μg/m^3^ per year (or 200 μg/m^3^ per hour on less than 18 days/year) (*16*). Compared to the United States (*3*, *17*), where NO_2_ and NO*_x_* have generally been declining steadily, current trends across urban European AQ networks show that NO_2_ has not decreased as projected (*14*, *18*). Because of slower than expected decrease in NO_2_ levels and stricter AQ standards, regulatory thresholds of NO_2_ are now violated at many stations (*19*). More recently, a slowdown of NO_2_ concentration reductions has also been reported over the contiguous continental United States (*15*), and speculation over the causes points toward several possibilities, including smaller than expected NO*_x_* reductions from selective catalytic reduction car exhaust systems. Significant primary NO_2_ emissions from internal combustion engines (ICEs), particularly Diesel engines (*20*), are also thought to play a major role. These uncertainties have far-reaching ramifications for air pollution control measures and have played a central role in the Diesel emission scandal (*21*). Modeling studies (*9*) have often relied on the assumption that ICEs primarily emit NO*_x_* in the form of nitrogen oxide (NO), but key questions in the context of urban NO_2_ pollution control remain: What fraction of NO*_x_* is directly emitted as NO_2_ (*9*), and to what extent does secondary partitioning within the O_3_-NO-NO_2_ triad ultimately control street canyon NO_2_ levels in high-NO*_x_* environments? Concerning primary NO_2_ emissions, fleet averages are particularly difficult to constrain. The reported range from individual exhaust plume and tailpipe measurements, for example, suggests that the NO_2_/NO*_x_* emission ratio can vary between 5 and almost 40% (*22*), depending on car make, driving conditions, emission standard, and vehicle age. The rapid interconversion within the O_3_-NO-NO_2_ triad has made it particularly hard to provide a quantitative top-down assessment of urban NO_2_ sources based on ambient concentration measurements.

Under sunlight conditions and high-NO*_x_* pollution, the cycling between the O_3_-NO-NO_2_ triad can be described by the following reaction sequence

$${\mathrm{NO}}_{2}+h\nu \to \mathrm{NO}+O$$ (1)

$$O+O_{2}\to O_{3}$$ (2)

$$\mathrm{NO}+O_{3}\to {\mathrm{NO}}_{2}+O_{2}$$ (3)

The chemical relaxation time scale to equilibrium of the NO*_x_* triad (Eqs. 1 to 3) (*23*) can be derived as

$$\tau =\frac{2}{\sqrt{\left[j^{2}+k_{3}^{2}{\left([O_{3}]-[\mathrm{NO}]\right)}^{2}+2j\cdot k_{3}([O_{3}]+[\mathrm{NO}]+2\cdot [{\mathrm{NO}}_{2}])\right]}}$$ (4)

where *j* represents the photolysis rate (Eq. 1) and *k*_3_ is the reaction rate constant defined by Eq. 3.

For typical midday summer conditions, this equates to time scales of about 100 s, comparable to the vertical turbulent exchange time in the urban boundary layer. Because of the rapid interconversion, the partitioning between NO and NO_2_ is typically dominated by chemistry, and inferences on primary NO_2_ emissions remain very uncertain. Furthermore, the rapid cycling between NO, NO_2_, and O_3_ is used to argue that steady-state conditions are achieved in mid- to high-NO*_x_* environments. This photostationary state (PSS) can be regarded as a null cycle and, according to Leighton (*24*), is defined by the following ratio (Φ)

$$\Phi =\frac{j_{{\mathrm{NO}}_{2}}[{\mathrm{NO}}_{2}]}{k_{3}[\mathrm{NO}][O_{3}]}$$ (5)

A number of studies evaluated this ratio (*25*–*27*) and reported Φ varying around one depending on the abundance of NO*_x_*. In more remote or rural regions, peroxy radicals (RO*_x_* = RO_2_ + HO_2_) can compete with reaction in Eq. 3, and deviations from PSS lead to Φ being systematically greater than one. This fact has been used extensively to constrain peroxy radicals in the atmosphere and test our current understanding of tropospheric ozone chemistry (*26*, *28*–*31*). More recently (*32*), it was argued that dynamical constraints were not fully considered in past assessments aiming to infer peroxy radicals from PSS. This fosters speculation whether the PSS approach generally under- or overestimates peroxy radicals. Incidentally, recent observations seem to point toward higher ozone production rates in high-NO environments (*6*). Could PSS have generally been underestimated? Since dynamical effects can lead to either negative or positive deviations from PSS, a conclusive answer whether past assessments were positively or negatively biased is currently not possible because of a lack of fundamental observational constraints.

The most direct approach for measuring turbulent surface fluxes is based on the eddy covariance method (*33*). Theoretical considerations (*34*–*37*) show that direct flux and concentrations measurements of the O_3_-NO-NO_2_ triad allow us to experimentally constrain the set of integrated equations (Eqs. 1 to 3). Here, we present long-term seasonal eddy covariance flux measurements to quantitatively test our current understanding of the urban O_3_-NO-NO_2_ triad in a high-NO*_x_* environment and combine these with remote sensing data and a theoretical model framework. By experimentally separating dynamical and chemical terms, assumptions inherent to the analysis of PSS can be tested in the context of chemical reactions and net import or export of NO, NO_2_, and O_3_. Furthermore, the fluxes of NO*_x_* (<*w*′NO′> + <*w*′NO_2_′>) and O*_x_* (<*w*′O_3_′> + <*w*′NO_2_′>) can be considered conserved and are proportional to the surface NO_2_ flux because the measured ozone flux directly represents the fraction of NO reacted to NO_2_ on transport time scales (e.g., 100 to 300 s) in the lowest part of the urban boundary layer. The presented observations are therefore uniquely positioned to shed light on the issue of primary city-scale NO_2_ emissions from combustion sources and the validity of PSS analysis in urban environments.

## RESULTS AND DISCUSSION

### Climatological overview of the urban NO-NO_2_-O_3_ flux dataset

**Fig. 1.**
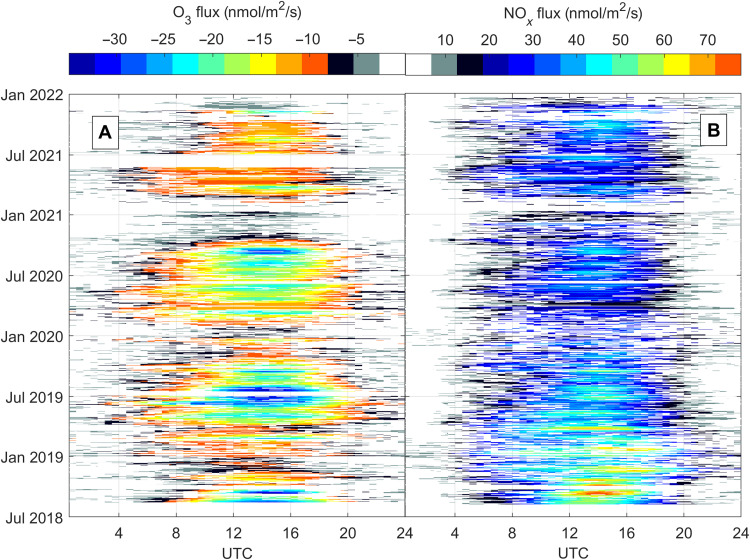
Plot showing diurnal variations of O_3_ and NO*_x_* fluxes. Climatology of O_3_ (**A**) and NO*_x_* (**B**) fluxes measured at the Innsbruck Atmospheric Observatory (IAO). Daily aggregated data are smoothed with a two-dimensional five-point running mean filter.

**Fig. 2.**
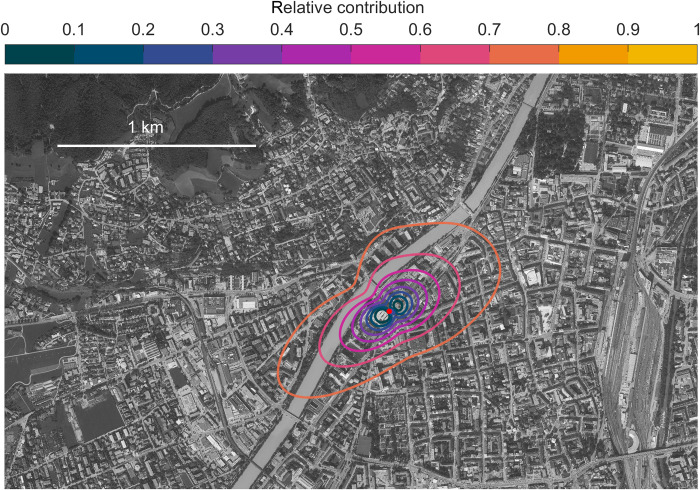
Flux footprint at the measurement site. The climatological flux footprint for the entire dataset at the surface site (isolines in 10% increments with the outer most isoline depicting the 80% flux footprint isoline) plotted on top of an orthomap (Orthophoto, Land Tirol, CC-BY). The emission flux (90%) is captured within the outermost isoline.

Figure 1 gives an overview of the underlying dataset used for this study. Observations reported here span nearly 4 years of continuously measured mixing ratios and fluxes of O_3_, NO, and NO_2_. Data shown in Fig. 1 are averaged with a two-dimensional five-point running mean filter. Along with maximum ozone mixing ratios [~70 parts per billion by volume (ppbv)], ozone fluxes exhibited clear minima during the summer. We observed minimum midday fluxes down to −60 nmol/m^2^ per second during the summer of 2018, when a European heat wave dominated the continent for several weeks. Typical NO*_x_* mixing ratios in Innsbruck are comparable to many other urban areas and can reach up to 150 ppbv during winter inversions. Measured average O_3_, NO_2_, and NO mixing ratios for the study period were 13.4, 19.6, and 9.7 ppbv, respectively. Typical daytime fluxes of NO*_x_* (NO_2_) lie between 15 and 45 nmol/m^2^ per second (10 and 22 nmol/m^2^ per second). For the summer 2018, when we complemented long-term measurements with additional meteorological and nonmethane volatile organic compound (NMVOC) observations, the average 24-hour flux of NO*_x_* and NO_2_ were 22 and 14.5 nmol/m^2^ per second, respectively. Because of the high-NO*_x_* environment, O_3_ mixing ratios are systematically low in the urban core and are often below the limit of detection during strong winter inversions. We previously demonstrated that NO*_x_* fluxes are dominated by traffic emissions (*18*) (i.e., >90% during daytime) and tend to be underestimated by bottom-up emission models due to unreported emissions. NO*_x_* fluxes (Fig. 1) typically follow the daily course of traffic patterns, comparable to previous results. The climatological flux footprint is depicted in Fig. 2 and shows the horizontal scale of surface emissions that are captured by measurements at the flux tower. NO*_x_* emissions are typically a factor of 2 to 3 lower on weekends than on weekdays. The weekend-weekday effect for the summer of 2018 shows that peak O_3_ was about 10 ppbv higher on weekends, reflecting lower NO*_x_* mixing ratios. This is comparable to what we observed in 2015, when the difference between weekend and weekday was on the order of 7 to 8 ppbv (*18*).

Surface in situ observations of NO_2_ toward the east agree to within the uncertainty of remote sensing observations obtained from a ground-based Pandora system, which has been used extensively to validate Tropospheric Monitoring Instrument (TROPOMI) observations (fig. S1, A and B). The TROPOMI monthly average for August 2018 shows a horizontal gradient of NO_2_ from east to west. This gradient is consistent with decreasing surface emissions toward the west. We can take advantage of these data to constrain daytime horizontal advection at the site. The average advection flux of NO_2_ was calculated from the horizontal NO_2_ concentration gradient inferred from TROPOMI, scaled to surface observations (*dC*/*dx* < 10^−4^ ppbv/m). The gradient was integrated from the displacement height (*d* = 18 m) to the measurement height (*z*_m_ = 42 m) and multiplied by the horizontal wind speed along the valley axis. Relative to the observed vertical flux of NO_2_, we find that the advection flux plays a minor role (i.e., <6% during daytime). Since, typically, 80 to 90% of daytime NO*_x_* exists in the form of NO_2_, the NO*_x_* advection flux is expected to be quite comparable to NO_2_. It corroborates conventional micrometeorological assumptions that daytime advection often plays a minor role, and we expect a similar behavior at other urban locations where NO*_x_* emission fluxes are comparable to observations shown here.

### Interpreting the urban Leighton ratio

**Fig. 3.**
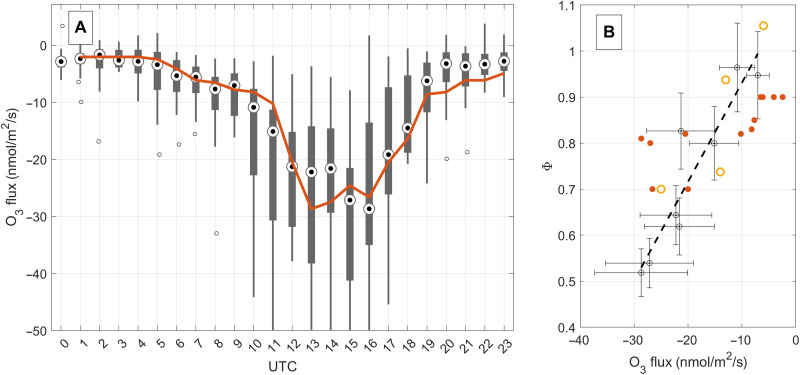
Diurnal variations of O_3_ fluxes and the dependence of the Leighton ratio on the O_3_ flux. (**A**) O_3_ flux. Data shown are for the summer 2018 during an intensive operational campaign. (**B**) Daytime Φ (i.e., Leighton ratio) versus O_3_ flux during the 2018 campaign (black/gray, observations; dashed line fit through 2018 observations; red solid dots, model). For comparison, all seasons are also plotted (orange open symbols), ranging from summer (lowest Φ), through spring, fall, and winter (highest Φ).

During daytime, we observed substantial downward fluxes of ozone (Fig. 1) and upward fluxes of NO_2_ and NO*_x_*. Ozone fluxes (Fig. 3) in the surface layer of the convective boundary layer (CBL) typically exhibited an absolute minimum around noon until early afternoon. Photochemistry is most active during summer, and Fig. 3 illustrates the detailed data analysis for the summer of 2018 when a large European heat wave persisted. During the heat wave, we used a number of additional observations (e.g., NMVOCs) as part of an intensive operational phase. Ozone exhibited median downward fluxes in the range of −20 to −30 nmol/m^2^ per second (minimum O_3_ flux, −62 nmol/m^2^ per second). A second-order closure model (*23*) of the O_3_-NO-NO_2_ triad can reproduce the diurnal cycle of ozone fluxes at the measurement height (~42 m) reasonably well. The corresponding “exchange velocity” (i.e., *v*_e_ = −flux/concentration) is 1 to 2 cm/s (maximum, 3.6 cm/s). The flux footprint (Fig. 2) shows that 90% of the surface is covered by buildings, roads, or concrete and only 10% is covered by vegetation for the data considered here. Under these conditions, a typical upper limit for a dry deposition velocity for ozone at the surface is on the order of 0.3 cm/s (see the Supplementary Materials), which is <20% of the observed O_3_ deposition velocity. A length scale analysis (fig. S4) demonstrates that the surface influence at 42-m measurement height is 0.17. The ozone flux at measurement height is therefore primarily (i.e., >96%, ~0.20 × 0.17) driven by chemistry, in particular, the reaction of NO + O_3_. Dahmköhler numbers are dimensionless quantities that describe the time scale of a reaction to that of transport and can be defined for mixing ratios and fluxes (see the Supplementary Materials). The Damköhler numbers and flux Dahmköhler numbers for ozone for the entire dataset are found to lie in the range of 0.1 to 0.8 and 0.2 to 1.2, respectively. They support the conclusion that both the flux and concentration profiles are significantly influenced by chemical conversion of NO to NO_2_. From an ecological perspective, these findings are quite relevant because such an efficient chemical conversion of ozone implies that urban vegetation is taking up O*_x_* in the form of NO_2_ rather than O_3_, which contrasts the situation of O*_x_* uptake in natural ecosystems, where plant exposure is dominated by O_3_ uptake. It has long been recognized that excessive ozone uptake is detrimental to plant health. On the other hand, it has also been shown that plants can quite effectively absorb NO (*38*) and NO_2_ (*39*) and use NO*_x_* as an alternative source of nitrogen, in addition to N fixation from soils. With projected future decreases in NO*_x_*, urban plants will gradually be exposed to more ozone. How much could that affect ozone exposure for urban plants? As a sensitivity experiment, we can look at the weekend/weekday ratio for the entire dataset. The yearly average NO*_x_* emission flux for weekdays is 22 and 10 nmol/m^2^ per second for weekends. The associated increase in ozone mixing ratios on weekends is ~10%. An established metric for the exposure of plants to ozone is the AOT40 (accumulated ozone exposure over a threshold of 40 ppbv). If we assume a future decrease in NO*_x_* emissions by a factor of 2 and a corresponding increase in ozone by 10%, we find that present-day AOT40 (5840 ppbv h) could increase to 9800 ppbv h, which would be quite significant.

Analogous to an enhanced spurious ozone deposition velocity, the Leighton ratio (Eq. 1) exhibited values close to 1 around 10 UTC but declined with increasing negative O_3_ flux. The ratio continued to drop over the course of the day, as the ozone flux became more negative. During conditions of low light (<6 and >18 UTC), the Leighton ratio becomes poorly constrained as *j*_NO_2__ approaches zero. These periods were therefore not used for the correlation plot in Fig. 3B. We find a decrease in the Leighton ratio with decreasing O_3_ fluxes. During midday and high-radiation conditions, we observe a Leighton ratio as low as 0.5 when the negative ozone flux peaked and is in the range of −25 to −30 nmol/m^2^ per hour. Within the uncertainty, the observed trends of vertical ozone deposition flux and Leighton ratio are generally reproduced by a simplified second-order closure model, which explicitly calculates second-order turbulence moments, with a predicted minimum of about 0.7. In urban areas, the establishment of the Leighton ratio is thought to be dominated by the O_3_-NO-NO_2_ triad due to high-NO emissions. To estimate the influence of RO*_x_* chemistry in the present study, we use a chemical box model sensitivity analysis that previously calculated a range of RO*_x_* between 2 and 15 parts per trillion by volume (pptv) (*18*). This is comparable to typically observed total peroxy radical densities (*40*). For a RO*_x_* mixing ratio of 8.5 pptv, the reaction rate of <NO + RO*_x_*> would be approximately 8% compared to the <NO + O_3_> null cycle reaction. Deviations of Φ < 1 are a direct consequence of NO diffusing upward reacting with O_3_. This will lead to a flux divergence of NO, NO_2_, and O_3_ (e.g., here *dF*_NO_/*dz* < 0), which, in turn, is the reason for a disbalance of the Leighton ratio. It will approach one when the flux divergence (*dF*/*dz*) approaches zero (e.g., top of the surface layer). The divergence of turbulent fluxes is caused by a disequilibrium of chemical destruction and production terms within the triad and potentially other chemical reactions (e.g., RO*_x_*). Mathematically, we can recast the Leighton ratio as following

$$\frac{\partial \bar{\left[\mathrm{NO}\right]}}{\partial t}=-\frac{\partial \bar{F_{\mathrm{NO}}}}{\partial z}+j_{{\mathrm{NO}}_{2}}\bar{\left[{\mathrm{NO}}_{2}\right]}-\bar{\left[\mathrm{NO}\right]}\cdot (k_{3}\bar{\left[O_{3}\right]})-k_{3}\bar{{\left[\mathrm{NO}\right]}^{{}^{\prime }}[O_{3}{]}^{{}^{\prime }}}$$ (6)

where the overbar represents a temporal average and the last part on the right side is the fluctuating part due to turbulent mixing. For simplification, let us assume that NO is in steady state, $(\frac{\partial \bar{\left[\mathrm{NO}\right]}}{\partial t}=0)$, and then it becomes clear that the deviation of Φ from 1 is driven by the vertical flux divergence term of NO: Surface NO emissions will drive this flux gradient. A significant downward flux of ozone (and upward flux of NO) then corresponds to a perturbation of Φ toward values below one. The produced fraction of NO_2_ is a direct consequence of the NO + O_3_ reaction and diffuses upward. In this context, we also constrained the influence of horizontal advection of NO_2_ using remote sensing data. We find that advection fluxes are minor compared to vertical turbulent fluxes of the NO-NO_2_-O_3_ triad. We find that a key process is how much NO is emitted and how much of ozone is transported downward as it reacts with NO driving a vertical flux gradient (i.e., divergence). While one might argue that measurements in a high-NO environment are dominated by the <NO + O_3_> reaction, it is clear that downward ozone fluxes also occur in areas where <NO + RO*_x_*> reactions can compete more substantially with the <NO + O_3_> reaction. Peroxy radicals inferred from the PSS approach therefore generally have to be considered to be biased low under most scenarios where there are sufficient NO surface emissions. We also investigated other seasons and find that ozone fluxes are smallest (i.e., highest) during winter (fig. S9), when peak daytime fluxes were about three to five times higher than during summer (e.g., −6 nmol/m^2^ per second in winter versus −30 nmol/m^2^ per second in summer). The reason is that winter ozone mixing ratios are typically more depleted because of excessive NO mixing ratios resulting in significantly smaller absolute ozone fluxes. In addition, convection is quite a bit smaller in winter than in summer. As expected, spring and autumn lie between the two other seasons. As a consequence, the Leighton ratio was, on average, close to 1 (1.05 ± 0.1) during winter (low–ozone flux season), while the seasonal average for summer lies around 0.7 ± 0.1 (high–ozone flux season), with the other seasons in between. Data presented here give a realistic range of the bias of the Leighton ratio as a function of ozone flux. The rapid conversion of NO to NO_2_ via reaction with O_3_ has a large impact on the formation of NO_2_ in the surface layer, making it hard to assess the direct amount of NO_2_ emitted from combustion engines, which is a relevant quantity for policy making.

### Primary emissions versus secondary production of NO_2_

**Fig. 4.**
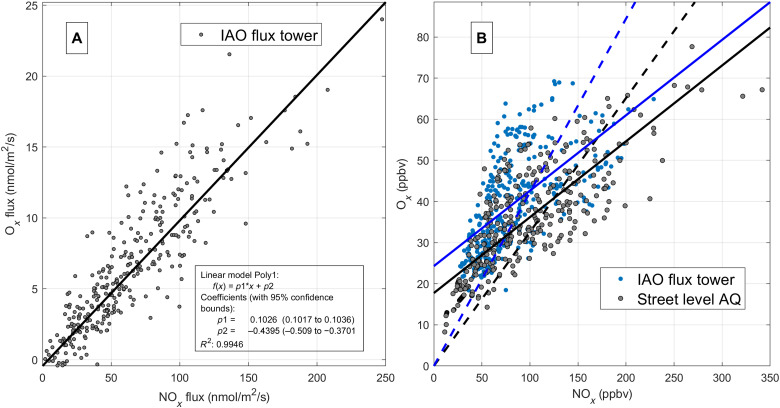
O*_x_* fluxes and mixing ratios plotted versus NO*_x_* fluxes and mixing ratios. (**A**) O*_x_* versus NO*_x_* fluxes at the flux tower for ambient O_3_ < 5 ppbv [slope = 10.3 ± 0.1 % and coefficient of determination (*R*^2^) = 0.99]. (**B**) O*_x_* versus NO*_x_* mixing ratios for ambient O_3_ < 5 ppbv at the flux tower (blue solid line; slope = 18.3 ± 2.5% and *R*^2^ = 0.38) and street level (black solid line; slope = 18.4 ± 1.5% and *R*^2^ = 0.62). Regressions with intercept forced through zero are 44 ± 1.6% (flux tower; dashed blue line) and 32 ± 1.0% (street level; dashed black line).

**Fig. 5.**
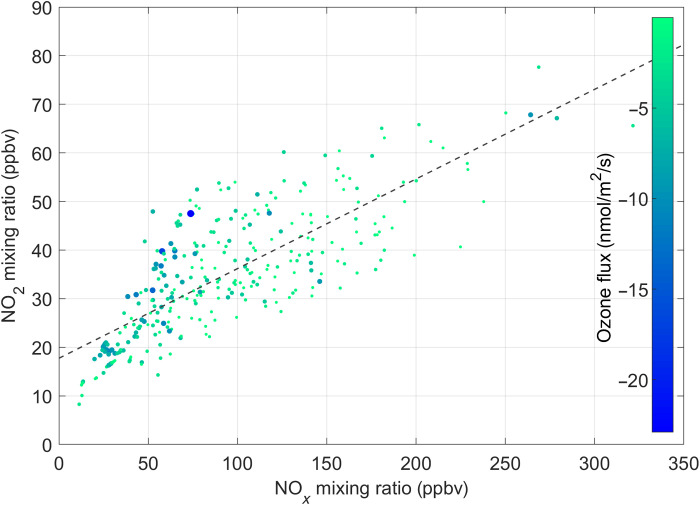
NO_2_ mixing ratios plotted versus NO*_x_* mixing ratios, color-coded by the vertical O_3_ flux. NO_2_ versus NO*_x_* mixing ratios for O_3_ < 5 ppbv at street level. Data are adjusted by size and color according to the vertical ozone flux measured at the flux tower. Fitted dashed line as in Fig. 4B.

**Fig. 6.**
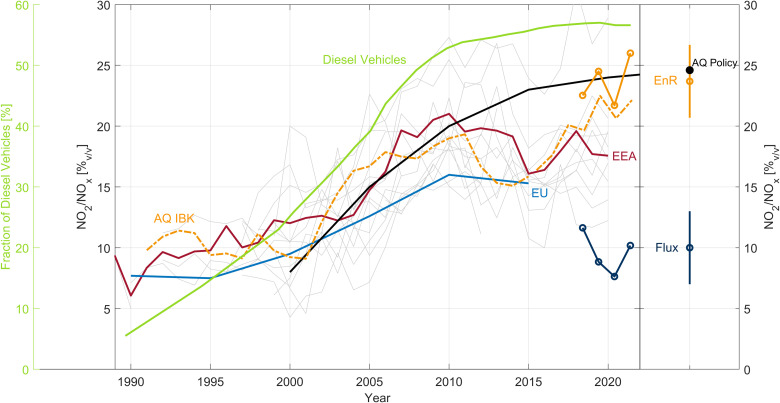
Fraction of Diesel vehicles in Austria plotted along with NO_2_/NO*_x_* EnR and flux ratiosRight Fraction of Diesel vehicles in Austria (left axis) plotted along with NO_2_/NO*_x_* EnR ratios: red line, ensemble average of selected European urban traffic AQ stations retrieved from European Environment Agency (EEA); gray lines, the individual ratios of the selected EEA traffic stations across Europe (including DE, UK AT, CH, and IT); light blue line, European average (*14*); black line, projection of European average (*19*); orange dashed line, urban traffic AQ station Innsbruck (IBK); orange circles, Innsbruck IAO EnR; blue circles, Innsbruck IAO flux ratio. (Right) Comparison between the EnR method (IAO; orange), the flux ratio method (IAO; blue), and AQ policy projections (same as black line) (*19*).

From a policy perspective, NO_2_ is considered a particularly relevant species due to its toxicity and ozone-forming potential. AQ thresholds are triggered for NO_2_ as a primary toxic air pollutant. This has sparked a debate on the effectiveness of reducing primary NO_2_ emissions to meet street-level NO_2_ pollution. For most combustion processes, the bulk of NO*_x_* is typically thought to be emitted as NO. Discrepancies between declining NO*_x_* and NO_2_ mixing ratios, however, have indicated that primary emissions of NO_2_ might play a significant role due to the emergence of Diesel vehicles (*22*, *41*). However, the magnitude of fleet average primary NO_2_ emissions has been particularly hard to constrain. Since primary emissions of NO will only convert O_3_ into NO_2_ due to the NO + O_3_ reaction, these emission fluxes would not have an impact on O*_x_* surface fluxes. However, direct emissions of NO_2_ should be detected in the O*_x_* surface emission flux. The overall lifetime of NO*_x_* is large enough (several hours) compared to the turbulent time scale (100 s) so that NO*_x_* can be regarded as a conserved tracer. As an example, Fig. 4 shows the relationships between O*_x_* (:=NO_2_ + O_3_) and NO*_x_* (:=NO_2_ + NO) fluxes (Fig. 4A) and mixing ratios (Fig. 4B) for the 2018 campaign. All data were preselected for ozone mixing ratios of <5 ppbv. The slope of the O*_x_* to NO*_x_* flux is directly related to the fraction of primary emitted NO_2_ at the street level. We find that the slope of O*_x_* versus NO*_x_* flux is 10.3 ± 0.1%. For concentration ratios, it is 80% higher (i.e., 18.4 ± 1.5% at the street level and 18.3 ± 2.5% at the flux tower). Looking more closely into the issue of secondary conversion, we find that, even at low ozone mixing ratios (<5 ppbv), the concentration ratio exhibits a dependence on the downward ozone flux. Figure 5 shows the NO_2_ versus NO*_x_* mixing ratios color-coded by vertical ozone fluxes measured at a close-by street canyon station. Data are filtered for O_3_ < 5 ppbv. Air masses that are systematically enriched in NO_2_ tend to be associated with higher downward O_3_ fluxes. We find a statistically significant difference between higher NO_2_/NO*_x_* ratios for larger negative ozone fluxes and lower NO_2_/NO*_x_* ratios for less negative (i.e., higher) ozone fluxes (even for O_3_ < 5 ppbv). On the basis of the two sample *t* tests, the hypothesis is rejected that the NO_2_/NO*_x_* ratio is independent of the ozone flux. We find that the mean bias at the 5% confidence level is 35% for NO_2_/NO*_x_* ratios below an ozone flux threshold of −5 nmol/m^2^ per second. The analysis is fairly independent of the chosen threshold but increases with lower thresholds as expected. It is, for example, 40% for an ozone threshold of −10 nmol/m^2^ per second. This suggests that low O_3_ mixing ratios (e.g., <5 ppbv) alone cannot guarantee that the conversion of NO to NO_2_ is negligible because of the distance between emission and detection, because higher ozone aloft can effectively mix down into the street canyon. The downward flux of ozone can therefore strongly modulate street canyon NO_2_ production. Incidentally, a recent study (*42*) has reported rapid interconversion of NO to NO_2_ at the tailpipe to curbside stage. Our measurements demonstrate that downward mixing and reaction of ozone must be the most important process driving the chemical conversion of NO to NO_2_ in urban areas. A primary NO_2_/NO*_x_* ratio of 10.3 ± 0.01% is significantly lower than inferred from more indirect approaches, for example, concentration enhancement ratios (EnR), which are affected by emission, chemistry, and transport of NO, NO_2_, and O_3_. It is also significantly lower than model projections (*19*) that were constrained to earlier indirect top-down approaches. Figures 4 and 5 illustrate that the interpretation of EnR of O*_x_* and NO*_x_* mixing ratios is quite a bit more complex than for direct fluxes, because various biases depending on data exclusion procedures can alter regression slopes for street canyon stations but tend to lead to an overestimation. Figure 6 extends the analysis to other years and locations based on commonly accepted data analysis approaches. We find that the bias is consistent across years. We also tested the sensitivity of the EnR method with respect to proximity to the emission source for Innsbruck. Data collected at the Innsbruck Atmospheric Observatory (IAO), about 42 m above street level, yield EnR ratios that are only about 16% higher than EnR data at a busy roadside AQ station near to the flux tower (e.g., orange dashed line versus orange circles in Fig. 6). While EnR data from Innsbruck yield comparable results to other European cities (Fig. 6 and see the Supplementary Materials) and previous policy assessments (*14*, *19*), we find that this approach overestimates the primary NO_2_ emission ratio by approximately a factor of 2 when compared to the more direct flux ratio method (10.3%), which explicitly accounts for the NO + O_3_ reaction.

### Implications for AQ policy assessments

The fraction of primary versus secondary emitted NO_2_ has consequences for predicting future improvements and trends of AQ. AQ trends in Europe show that NO*_x_* and NO_2_ mixing ratios have not declined as expected from past assessments (*16*) and that NO_2_ mixing ratios declined less rapidly than NO*_x_*. This has brought many cities out of compliance due to stricter AQ limits for NO_2_ (40 μg/m^3^), which were implemented according to WHO guidelines (*13*) and were based on optimistic projections of declining NO*_x_* and NO_2_ emission trends. We investigated the magnitude of this effect for the present high-NO*_x_* location. On the basis of Eqs. 1 to 3, it follows (see the Supplementary Materials) that a change in urban NO_2_ trends due to changing NO*_x_*, O_3_, temperature, and photolysis rates can be related according to

$$\frac{d[{\mathrm{NO}}_{2}]}{\left[{\mathrm{NO}}_{2}\right]}=\frac{d[{\mathrm{NO}}_{x}]}{\left[{\mathrm{NO}}_{x}\right]}+\frac{j\;dm}{m(m+j)}-\frac{1}{j+m}dj$$ (7)

where *j* := *j*_NO_2__ and *m* := *k*_3_ * [O_3_].

Similar to many European cities, NO*_x_* mixing ratios in Innsbruck have been declining since about 2004. An annual trend for the years between 2004 and 2016 was calculated using in situ data obtained from nearby AQ stations (*43*). The predicted NO_2_ trend [i.e., δ(NO_2_)/(NO_2_)] based on Eq. 7, taking into account changes in NO*_x_*, O_3,_ temperature, and photolysis rates, is −1.7% per year. The individual contributions (Eq. 7, right-hand side) are −2.2% (term 1), +0.5% (term 2), and −0.004% (term 3), respectively. The actually measured annual NO_2_ trend was higher (i.e., −1.9% per year) than that of NO*_x_* (−2.2%). While this difference could potentially be attributed to an increasing primary NO_2_ emission fraction, we find that the effect of increasing ozone mixing ratios (term 2, 0.5%) over the same time period can also explain the difference on an annual basis. As shown here, the downward mixing of ozone plays a major role for urban NO_2_ mixing ratios. We find that the individual budget terms (Eq. 7) are internally consistent within the uncertainty. While our analysis supports the commonly accepted view that there is a contribution of primary NO_2_ emissions from Diesel-fueled exhaust, we find that the absolute fraction is likely overestimated. In Austria, the fraction of Diesel passenger vehicles has increased from less than 10% before 1985 to 55% in 2018 (Fig. 6). On the basis of NO_2_/NO*_x_* EnR trends calculated for representative road-side and inner city locations across Austria and compared to Europe wide assessments, the fraction of primary NO_2_ emissions has increased along with the fraction of Diesel vehicles. Trends have recently leveled out, and we argue that this is primarily due to a stabilization of the fraction of Diesel vehicles in conjunction with readjusting ozone mixing ratios. Diesel engines tend to emit a higher fraction of NO*_x_* in the form of NO_2_ compared to gasoline vehicles (*44*). While NO*_x_* emissions generally increase over the vehicle’s lifetime (*45*), the fraction of primary NO_2_ is thought to decrease (*14*). The combined effect for an entire vehicle fleet is therefore particularly hard to asses. Direct flux measurements, reported here, show that the amount of primary fleet average NO_2_ emissions is substantially lower than estimates from past policy assessments (*19*) and even lower than more recent indirect analysis methods (i.e., by 80%; Fig. 6). Adjusting EnR trends according to direct flux data yields a fraction of NO_2_/NO*_x_* that has stabilized at a level of 10%. By taking this ratio and a NO_2_/NO*_x_* emission ratio of 2 to 5% for gasoline vehicles (*46*, *47*), we obtain a primary NO_2_/NO*_x_* emission ratio of about 15 to 18% (by volume) for the current average Diesel fleet in Europe.

Direct flux observations of the O_3_-NO-NO_2_ triad show that the downward flux of ozone into the urban roughness layer is dominantly driven by the rapid conversion of NO to NO_2_. We find that 90% of urban NO_2_ production is due to the chemical conversion within the triad. On the scale of air chemistry models used for policy making, the downward ozone flux above urban areas is therefore largely controlled by NO emissions, which become an important factor for subgrid-scale deposition parameterizations of O*_x_*. As a consequence, urban plants absorb most O*_x_* in the form of NO_2_ rather than O_3_, which is considered detrimental to plants. This contrasts deposition in natural habitats. We find that a future reduction of NO*_x_* emissions by a factor of 2 can increase AOT40 levels for urban vegetation by 67%. The rapid chemical conversion also has a profound impact on the Leighton ratio, which is significantly modified and can be biased low in high-NO environments. Depending on the ozone flux, peroxy radicals estimated from the photochemical steady state will therefore always be underestimated at the surface and might require a reinterpretation based on findings presented here. These observations support recent studies (*6*) that seem to point toward higher ozone production rates when NO emissions increase. We also find that a substantially smaller than expected fraction (i.e., 10%) of NO_2_ is directly emitted into the urban atmosphere and attribute a fleet average primary NO_2_/NO*_x_* emission ratio for Diesel vehicles to be on the order of 15 to 18% in Central Europe. NO*_x_* emissions, however, are known to have been significantly underestimated by recent emission inventories across European metropolitan areas and elsewhere because of unreported emissions (*18*, *21*, *48*). Our analysis demonstrates that street canyon NO_2_ mixing ratios are ultimately controlled by NO emissions and the vertical ozone flux, which also perturbs the photochemical state. We show that NO_2_ mixing ratios have been declining less rapidly than NO*_x_*. The discrepancy can be explained by increasing O_3_ mixing ratios over the same time period, suggesting that increasing ozone mixing ratios in the future can lead to more efficient secondary conversion of NO to NO_2_ in street canyons, slowing the decline of urban NO_2_. Attainment of NO_2_ in urban areas will therefore largely remain a function of NO emissions and ozone entrainment fluxes from the free troposphere into the urban boundary layer.

## MATERIALS AND METHODS

### Site description

The eddy covariance flux tower was located at the IAO (latitude, 47°15′51.66″; longitude, 11°23′06.82″) in proximity of two street canyon AQ stations (AQ_F: latitude, 47°15′45.5′N; longitude, 11°23′32.5″E; AQ_A: latitude, 47°16′16.7″N; longitude, 11°25′01.0″E). Data from these stations were used to assess street level EnR. A detailed description of the flux tower was previously published (*18*). A flux tower was constructed at the southeast corner of an eight-floor university building in 2017. The height of the upper trace gas inlet and sonic position was determined from high-resolution laser scan data (Land Tirol) and was 41.2 ± 1.1 m above the mean street level. The dominant wind direction at the site is represented by a characteristic valley flow regime (see the Supplementary Materials). The general wind direction followed the expected valley wind system where most of the flux data for sensible heat, trace gases, and aerosols are captured along the northeast (~60°, ~33% of the time) and southwest (~220°, ~41% of the time) valley axis. The northeast (40° to 90°) corridor captures most of the inner city of Innsbruck and is representative of a typical urban fingerprint. The southwest sector (160° to 260°) represents mostly an urban residential area.

For measurements presented here, we constrain the analysis to the east sector that dominates during daytime. It also reflects the direction toward the urban AQ stations operated by the state of Tirol (47°15′45.5″N, 11°23′32.5″E). The mean building height in the east sector was calculated from high-resolution laser scan data provided by the state of Tirol (Land Tirol). The distribution of mean building height within the footprint of the east sector was calculated as *z*_h_ = 18.2 ± 2.4 m.

### Instrumentation

A dual channel chemiluminescence instrument (CLD 899 Y, Ecophysics) was used for high-frequency NO and NO*_x_* measurements. The instrument was operated in a fast mode acquiring data at about 5 Hz. A NO standard was periodically introduced for calibration. Zeroing was performed once a day close to midnight. Damping time scales for eddy flux measurements were determined as 0.8 s.

Direct NO_2_ measurements were also conducted using a cavity ring-down spectrometer (CARDS) (Los Gatos Inc., USA). The instrument was operated in a fast mode with a cell pressure of about 300 torr. The sample flow was predried with a Nafion dryer. Periodic zeros through a NO_2_ scrubber were performed every 180 min. The direct comparison of NO_2_ mixing ratios between CARDS and nCLD 899 Y shows a good correlation [slope = 0.94 ± 0.01 and coefficient of determination (*R*^2^) = 0.98], suggesting minimal impact of NO*_y_*-interfering species for the chemiluminenscence method (*49*).

High-frequency ozone measurements were obtained from a closed-path fast-response chemiluminescent instrument (Sextant Technology, NZ), which was calibrated using parallel measurements from a closed-path ultraviolet (UV) photometric analyzer (APOA-360, Horiba, Japan). A second closed-path UV photometric analyzer was operated at street level. Chemical measurements at the tower were drawing sample ambient air from a pressure-controlled turbulently purged 3/8″ Teflon line, with an overall lag time of about 1 to 2 s. Calibration procedures for dry chemiluminescence methods based on coumarin have been devised previously (*50*). A closed path eddy covariance system (CPEC 200, short inlet, enclosed IRGA design, Campbell Scientific) measured three-dimensional winds along with CO_2_ and H_2_O. Calibration for CO_2_ was performed once a day. A four-channel net radiometer was housed on the flux tower. A second CPEC eddy-covariance system was deployed at street level. AQ data of NO, NO_2_, and O_3_ were obtained from two nearby AQ stations.

Application of eddy covariance in urban areas have been assessed extensively (*51*) and were calculated as the covariance between the rotated vertical wind speed and the tracer mole fraction using routines described previously (*33*). Quality control was performed according to procedures described by Foken and Wichura (*52*). Systematic errors due to high-frequency losses were obtained from cospectral analysis according to Massman and colleagues (*53*).

### Models

SOMCRUS (Second-Order Model for Conserved and Reactive Unsteady Scalars) is a one-dimensional second-order closure numerical model to study the vertical turbulent transport of trace reactive species in the (daytime) planetary CBL. The model includes basic reactions of the O_3_-NO-NO_2_ triad. The Master Chemical Mechanism (MCM) developed by the National Centre for Atmospheric Sciences at the University of Leeds summarizes the state-of-the-art knowledge on tropospheric chemistry. The chemical mechanistic information was taken from the MCM v3.3 via the website: http://mcm.york.ac.uk/.

## Supplementary Materials

### This PDF file includes:

Supplementary Text Figs. S1 to S9 References
