# Supplementary material for: High urban NOx triggers a substantial chemical downward flux of ozone
Source: Sci Adv. 2023 Jan 18;9(3):eadd2365. doi: 10.1126/sciadv.add2365 (PMC9848777; doi:10.1126/sciadv.add2365)
Supplement: Supplementary file 1 — Supplementary Text Figs. S1 to S9 References [file sciadv.add2365_sm.pdf]

Science Advances
AAAS

Supplementary Materials for

High urban NO_x triggers a substantial chemical downward flux of ozone

Thomas Karl *et al.*

Corresponding author: Thomas Karl, thomas.karl@uibk.ac.at

Sci. Adv. **9**, eadd2365 (2023)
DOI: 10.1126/sciadv.add2365

This PDF file includes:

Supplementary Text
Figs. S1 to S9
References

Supplementary Information

Remote Sensing Data

TROPOMI column measurements

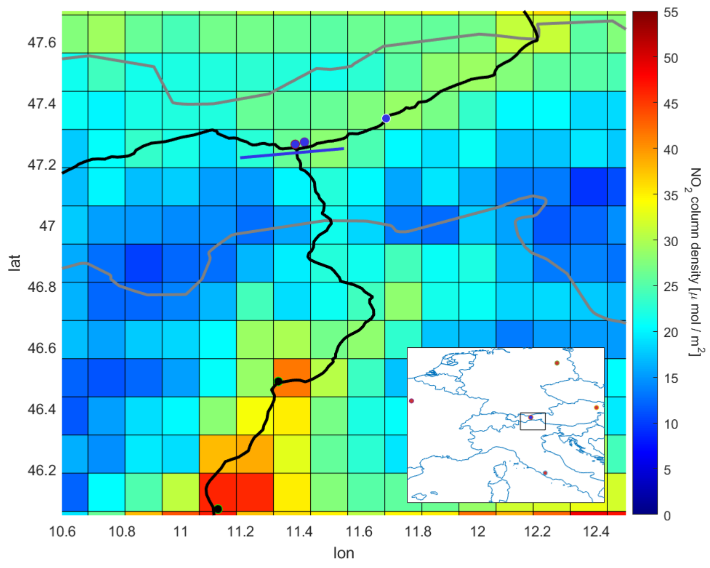

Figure S1a: Tropomi climatology for 2018. Black lines: major highways (A13, A12, E45, A22). Grey lines: Austrian borders. Blue dots: measurement locations in Innsbruck (IAO), and AQ stations in Innsbruck and along the A13. Black dots: regional cities of Bozen and Trento. Insert: larger view of study area (black box) in Central Europe (borders: blue lines)

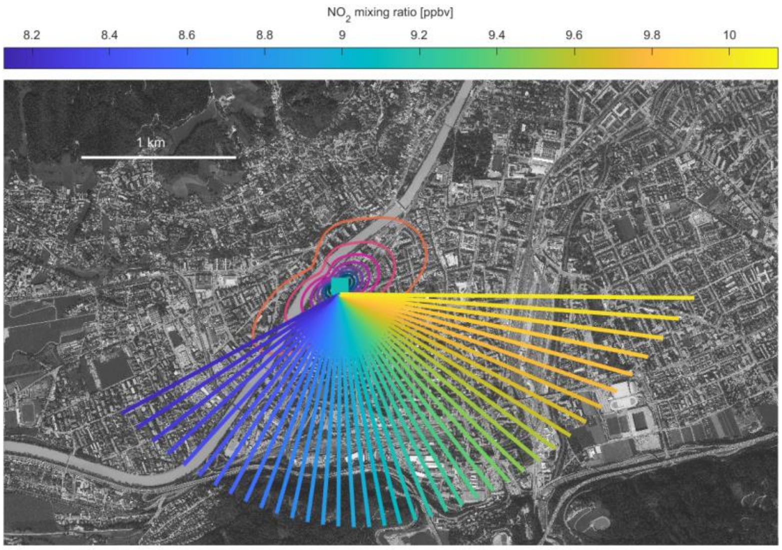

Figure S1b: Daytime NO2 mixing ratios measured during 2018 campaign. Square symbol represents mean mixing ratio at IAO from in-situ measurements. Line trajectories are the

computed mean PBL concentrations from Pandora, which follows the sun. The decrease of Pandora mixing ratios is a consequence of diurnal and spatial variation.

Flux Footprint

The flux footprint (Fig 2.) was calculated according to Kljun et al. (*54*). A climatology of the land surface distribution for 2018 is depicted in Fig. S2. Within the flux footprint the average amount of vegetation was on the order of 20%. Relevant for the analysis of ozone fluxes is the sector between 55 and 120 °, with a contribution from vegetated surfaces of about 10%.

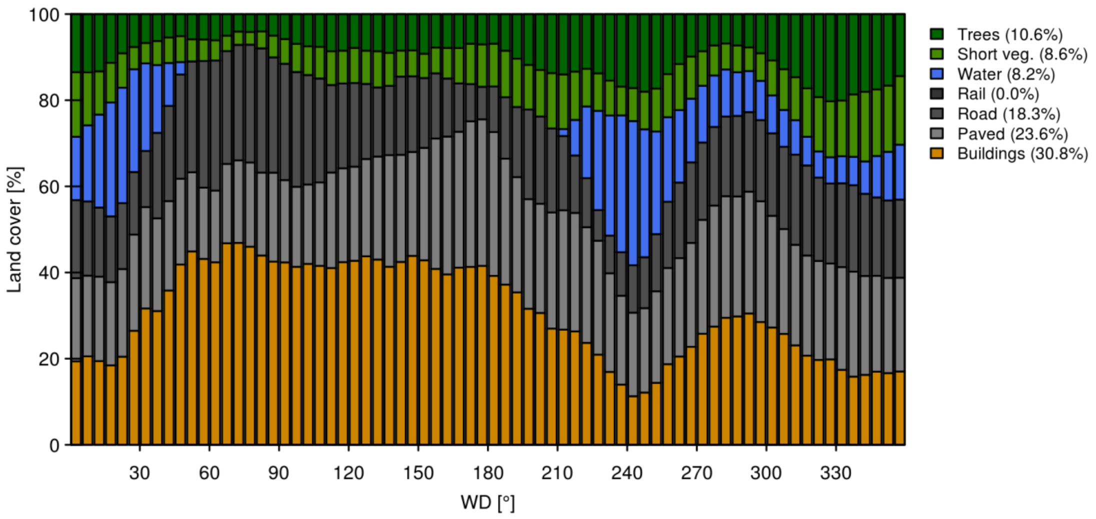

Figure S2: Land cover distribution within the 90% footprint. Relevant for the analysis is the sector between 55 and 120 °

Dry Deposition

An upper limit for dry deposition of ozone at the surface was calculated according to Wesely (*55*). We apportioned the surface deposition flux according to the flux footprint, showing that 90% consists of typical urban surface elements (e.g. buildings, concrete, roads) and 10% is vegetation. To estimate an upper limit for dry deposition we assumed lush vegetation. A Matlab code for calculating dry deposition rates is provided separately.

Damkoehler numbers

The Damkoehler number for ozone was calculated according to

$$Da_{O3} = \frac{z_m}{u^*} \cdot k \cdot [NO], \text{ (eq. S1)}$$

where k is the reaction rate constant for the reaction NO and O_3, z_m is the measurement height and u* is the friction velocity. Damkoehler numbers for NO and NO2 are defined similarly, taking their reaction rates into account.

The flux Damkoehler number for ozone is defined according to :

$$Da_{flux}(O_3) = \left| Da_{O3} + Da_{NO}\frac{F_{NO}}{F_{O3}} - Da_{NO2}\frac{F_{NO2}}{F_{O3}} \right|, \text{ (eq. S2)}$$

where F_x represent the measured fluxes for O_3, NO and NO_2, and Da_x are the Dahmköhler numbers for O_3, NO and NO_2 respectively.

Comparison of different indirect experimental approaches for estimating primary urban NO_2:

Two indirect methods can be used to infer enhancement ratios (EnR) and flux ratios of NO_2 to NO_x. The most common way is based on conventional regression methods that aim to linearly fit NO_2 vs NO_x after filtering data based on empirical methods (e.g. by choosing periods of low ozone at regional AQ background stations) (*56*). Due to the local imbalance between emission, chemistry and transport terms including the influence of turbulence on chemistry (*57*) biases can be introduced. The main bias that is usually considered is the fast interconversion within the triad. Due to the NO + O_3 reaction most of the NO_2 is produced secondarily in urban areas. This effect can lead to an overestimation of primary NO_2. It is observed for any urban NO_2 vs NO_x correlation plot. To demonstrate this effect, we use data from an urban AQ station situated at a busy traffic intersection downtown in Innsbruck. Data for the year 2018 were pre-filtered using only ozone concentrations below <10 ppbv. Fitting slopes through the two end member distributions results in slopes between 0.65 and 0.18. The upper limit must clearly be influenced by secondary production of NO_2. Common data filtering methods aim to select periods when the secondary production of NO_2 due to the NO+O_3 reaction exerts a minimal influence.

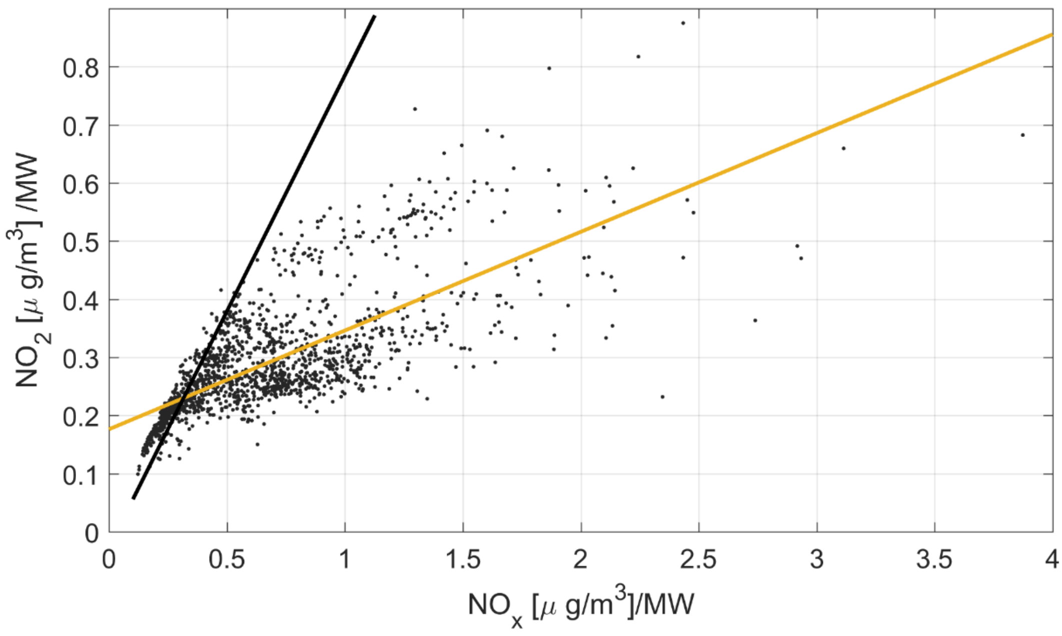

Figure S3: Regression of NO_2 over NO_x using data from an AQ station for O_3 <5 ppbv in 2018. The black line is fitted through the lower range of data, the orange line is a fit through all data.

These regressions can be compared to direct flux measurements of O_x vs NO_x, which can be regarded as a physically first principle benchmark. These flux data suggest a primary NO_2 to NO_x

ratio of 0.10±0.02 in 2018. Fitting hourly AQ NO_2 over NO_x data to obtain primary NO_2 emission ratios harbors several pitfalls, which can result in various compensating or amplifying biases. First, the non-linear chemistry of the O_3-NO-NO_2 triad results in significant secondary production of NO_2, which generally results in an NO_2/NO_x ratio that is 2-4 times higher than the primary emission ratio. Filtering techniques of concentration data are not always well defined to estimate the secondary production term. Second, by filtering for periods of regional low surface ozone it is assumed that ozone and NO_x concentrations aloft, which effectively entrain into the street canyon, are identical to these surface concentrations. When the slope becomes small we find that the regression analysis using concentration data is particularly sensitive to random errors and large offsets inherent to any ambient concentration dataset. Our analysis suggests that care must be taken when interpreting NO_2/NO_x correlation plots to infer primary NO_2 ratios. Flux data suggest that the vertical entrainment of ozone into the street canyon is essentially governing the influence of a systematic overestimation of NO_2 to NO_x concentration ratios obtained at street canyon air quality stations.

A second indirect approach (F_{NOx} method) to estimate primary urban NO_2/NO_x emission ratios can be based on direct flux measurements of NO_2 and NO_x, but without knowledge of O_3 fluxes. Without knowledge of ozone fluxes this approach relies on knowledge of the secondary formation of NO_2 from flux divergence terms. An analytical (theoretical) solution for the NO to NO_2 flux conversion based on the reaction-diffusion equation was first derived by Lenschow and coworkers (*23*). They found that the flux of NO or NO_2 can be expressed as:

$F\left(\frac{z}{l_1}\right) = 2\sqrt{\frac{z}{l_1}} \cdot BesselK_1\left(2\sqrt{\frac{z}{l_1}}\right)$ (eq. S3), where

z is the height above ground, $BesselK_1$ is the modified Bessel function of the second kind. l_1 is the vertical scaling length, which can be expressed as a function of friction velocity (u*), van Karman constant (k=0.4), reaction rate constant k between NO and O_3 (see eq.1), mean ozone mixing ratios (assumed constant over height z at the geometric mean), and the photolysis rate for NO_2 (j):

$l_1 = \frac{u^* k}{k_3 \cdot [O_3] + j}$ (eq. S4).

With increasing height, errors become large (ie. $z/l_1 > 1$) and this method bears several pitfalls for estimating primary NO_2/NO_x emission ratios due to systematic uncertainties entering the correction terms through eq. S4. Fig. S4 shows the effect of the correction term that has to be applied to estimate primary NO_2/NO_x emission ratios from NO2 and NOx flux measurements at the flux tower measurement height in Innsbruck. Despite these shortcomings we find that this method leads to a primary NO_2/NO_x ratio of 0.11±0.05 for Innsbruck. This is quite comparable to the direct approach discussed in the main manuscript.

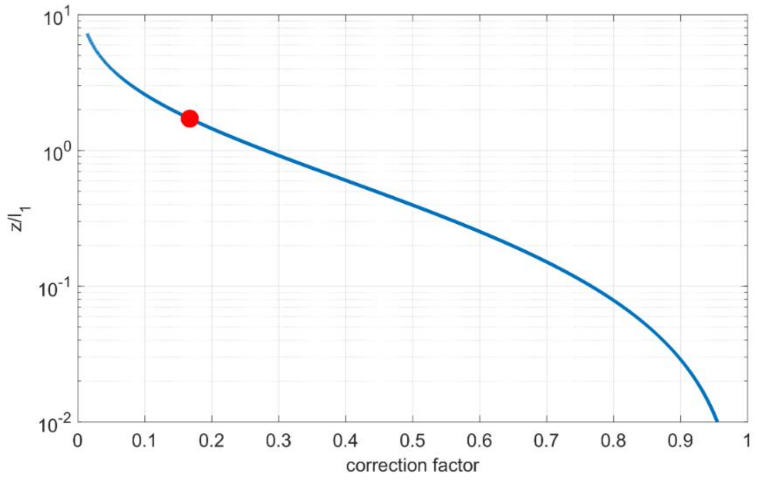

Figure S4: Secondary production of NO_2 displayed as correction factor as a function of measurement height according to (eq S4). The red circle represents typical conditions for the Innsbruck urban flux tower (IAO)

Modeling framework and uncertainty analysis

Figure S5 shows the general modeling framework. SOMCRUS (Second Order Model for Conserved and Reactive Unsteady Scalars) (*34*) is a one-dimensional second order closure numerical model to study the vertical turbulent transport of trace reactive species in the convective (daytime) planetary boundary layer (CBL). The model includes explicit reactions of the O_3 – NO – NO_2 triad. It does not include hydrocarbon chemistry. The model was initialized using measured sensible heat, NO, NO_2 and O_3 fluxes at 40 m which were extrapolated to the surface. Here, we primarily use the model to interpret O_3 – NO – NO_2 triad fluxes and concentrations in the urban surface layer, where rapid cycling within the triad dominates due to the high NO environment. In order to account for a net import of ozone produced above the CBL (e.g. via transport and chemical production), concentrations entraining into the mixed layer were set to NO = 0.7 pptv, NO_2 = 3.7 ppbv, and O_3 = 65 ppbv. The CBL height (h) was calculated using the Tennekes model for a free-convection mixed-layer (ML) that incorporates the surface buoyancy flux, an initial mixed-layer height of 200 m, a free troposphere (FT) lapse rate of 12 K km^{-1}, and a divergence of 10^{-6} s^{-1}. These values were adjusted to give a mid-day CBL height of about 1 km as observed. In a sensitivity run the FT lapse rate was adjusted from 12 K km^{-1} to 8 K km^{-1}, and a displacement height of 18 m was incorporated. The divergence was changed from 10^{-6} to 1.5 x 10^{-6} s^{-1}. This gives a late afternoon h of 1.050 m. For the fast cycling between NO, NO_2 and O_3, RO_x chemistry plays a minor role and the triad cycling dominates, while RO_x +NO certainly contributes to ozone production aloft on timescales limited by the reaction of hydrocarbons + OH. As mentioned above SOMCRUS runs did not include explicit hydrocarbon chemistry. Instead the net import of ozone into the urban surface layer was parameterized using observed ozone concentrations at 40m (ie. by adjusting entrainment rates and background ozone concentrations). Model outputs are solely used to interpret the effects of fast triad cycling with respect to surface fluxes and fluxes measured at the 40 m height.

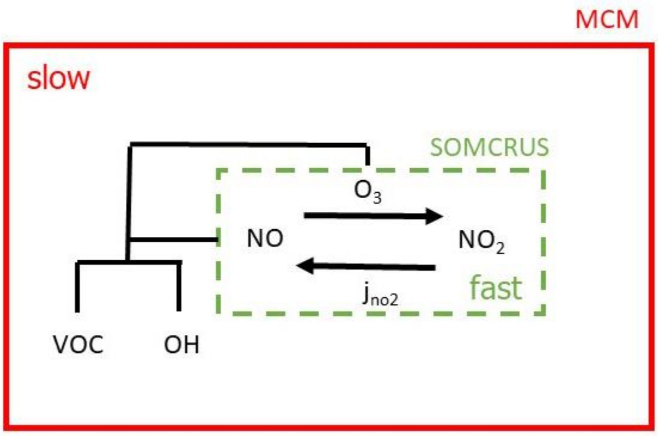

Figure S5: Conceptional modeling framework: Fast chemistry of triad concentrations and fluxes is constrained by SOMCRUS. The sensitivity of slow chemistry is modelled by a near explicit chemical box model based on the master chemical mechanism.

Effects of slow chemistry are considered using the MCM v3.3 as a boxmodel constrained by surface observations. The Master Chemical Mechanisms developed by the National Centre for Atmospheric Sciences at the University of Leeds summarizes the state of art knowledge on tropospheric chemistry. The chemical mechanistic information was taken from the Master Chemical Mechanism, MCM v3.2 via the website: http://mcm.york.ac.uk/, and processed for further analysis in Matlab (www.mathworks.com) using the box model version of CAFÉ (*58*, *59*). The model was initiated with measurements of VOC, CO, CH4, NOx, O3 and radiation and run for 10 minutes to let radicals (OH, HO_2 and RO_2) evolve to quasi steady state. This approach as been used in the past extensively to constrain atmospheric chemistry models (*60*). Fig. S6 to S7 show the quasi steady state calculations for OH and HO_2 + RO_2. A sensitivity study was performed to change NO_x and VOC concentrations over a wide range.

Uncertainty analysis:

Other chemical reactions in the boundary layer

On timescales of boundary layer dynamics considered here (e.g. 10-20 min) uncertainty effects arising from secondary (ie. slow) chemistry not included in SOMCRUS were estimated using additional measurements constraining a 0-D box model (MCM) (Fig S6 to Fig. S7). From this analysis, the NO_2 lifetime due to reaction with OH is estimated to be on the order of 5.7h. The timescale of PBL mixing based on measurements and modeling (h/w) is on average 600 s (450 - 919 s)). We therefore conclude that on timescales of PBL mixing (450 - 919 s), additional loss effects for NO_x over the entire PBL are on the order of 3-5%. Since we use SOMCRUS outputs primarily for the interpretation of surface fluxes, the chemical loss of NO_x is likely to be lower. For comparison the scale of PBL growth and entrainment, which is explicitly modelled by SOMCRUS, yields an average daytime timescale of 22h and corresponds to a dynamical mixing loss for NO_x on the order of 25% during midday.

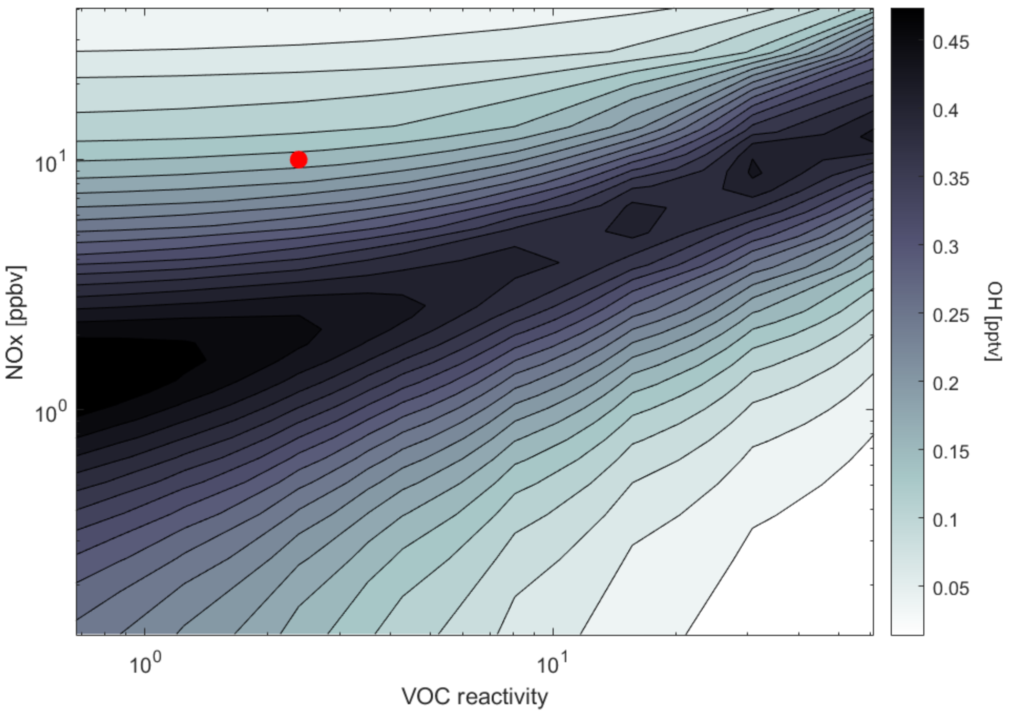

Figure S6: Isopleth plot of OH. Red dot indicates conditions for Innsbruck

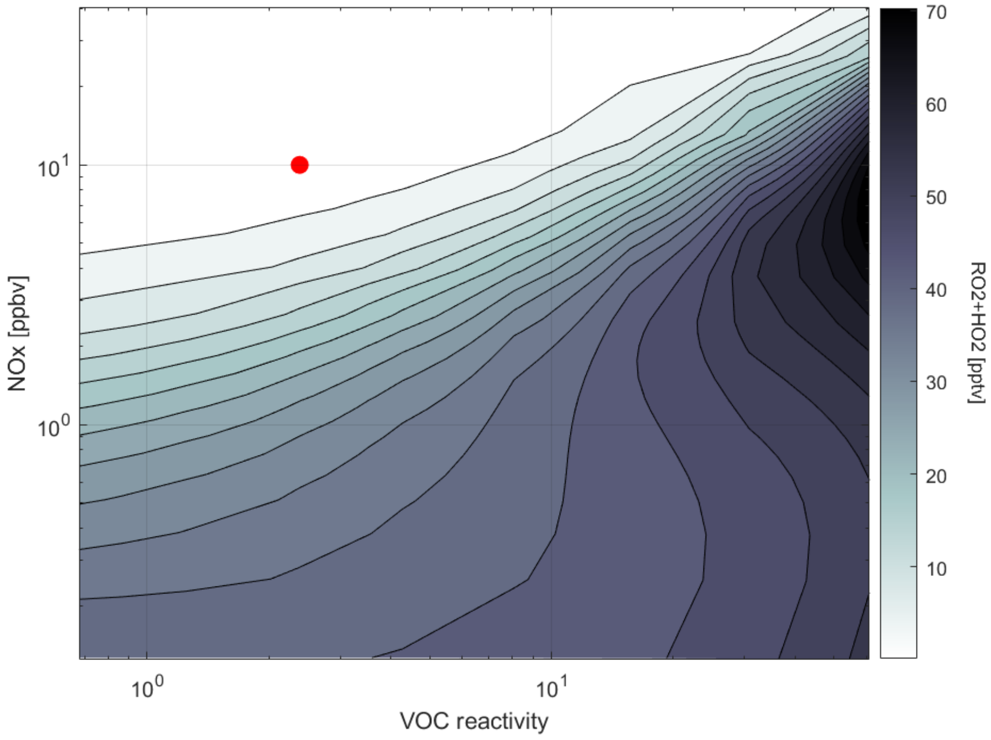

Figure S7: Isopleth plot of RO_x. Red dot indicates conditions for Innsbruck

Chemiluminescence NO2

Several NO_y species have been identified as potentially interfering species for chemiluminescence measurements. In order to investigate these biases in more detail for Innsbruck, results from fast chemiluminescence measurements were previously compared to a direct NO2 method, based on cavity ring down spectroscopy (*18*). We find a potential uncertainty on the order of 5-6%.

Comparison of PBL height and growth using SOMCRUS and a parameterization

A validation of estimated boundary layer (PBL) height was conducted for the summer field campaign in 2018. We used SOMCRUS to calculate PBL heights for this campaign (*34*). In addition we calculated PBL heights based on a parameterization using surface heat fluxes (*61*). For 2018 we also have PBL height estimations from a LIDAR system. A theoretical LES modeling study based on idealized topography for Innsbruck was previously conducted by Leukauf et al. (*62*) . We used their estimates for a fully developed unstable PBL. All approaches are shown in Fig. S8.

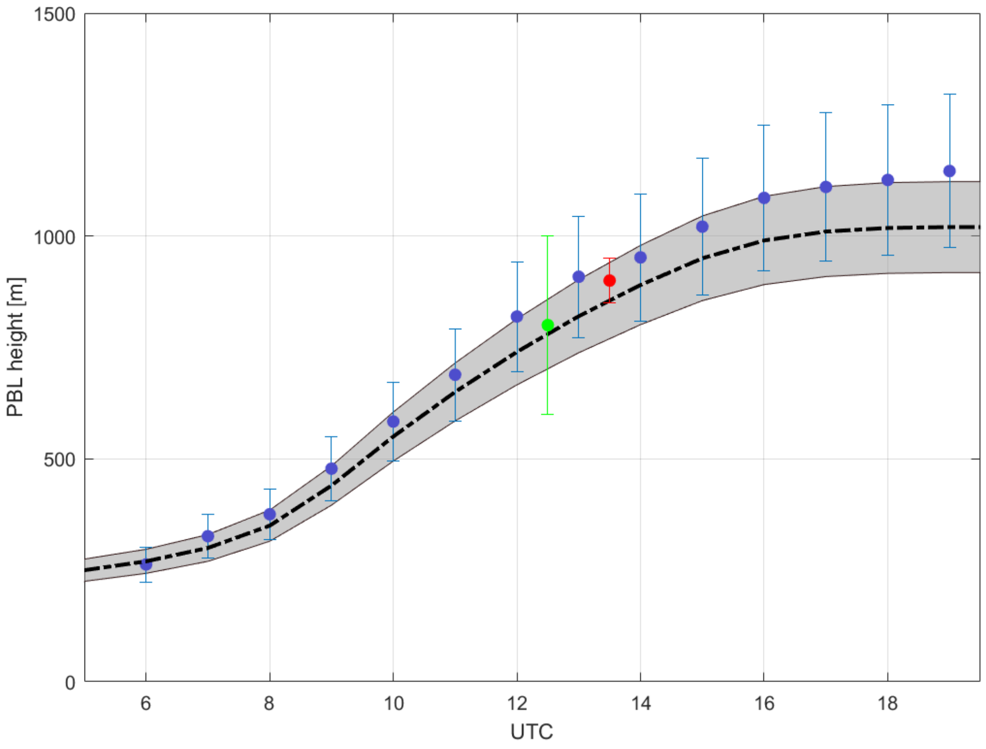

Figure S8: PBL estimates based on four approaches: SOMCRUS (black dotted line and model uncertainty in shading), wT parameterization (blue dots), LIDAR 2018 campaign (green dot), theoretical LES study (red dot)

Derivation of equation 7

Let $NO_x = NO_2 + NO$, and the Leighton ratio be defined as:

$$\Phi = \frac{j_{no2}[NO_2]}{k_3[NO][O_3]} \text{ (eq. S5)}$$

For simplicity we define m = k_3 * [O_3], and j=j_{no2} then

$$[NO_2] = \frac{[NO_x]}{(1+\frac{j}{m})} \text{ (eq. S6)}$$

The sensitivity of d[NO_2]/NO_2 can be simply obtained by first calculating the natural logarithm on each side:

$$ln([NO_2]) = ln([NO_x]) - ln\,(1+\frac{j}{m}) \text{ (eq. S7)}$$

then calculating the derivative of each quantity e.g. for the left side:

$$\frac{\partial ln[NO_2]}{\partial [NO_2]} \cdot d[NO_2] = \frac{d[NO_2]}{NO_2} \text{ (eq. S8)}$$

For the right side we obtain:

$$\frac{dNO_x}{NO_x} + \frac{j\,dm}{m(m+j)} - \frac{1}{j+m}dj \text{ (eq. S9)}$$

Comparison of ozone fluxes during the seasons

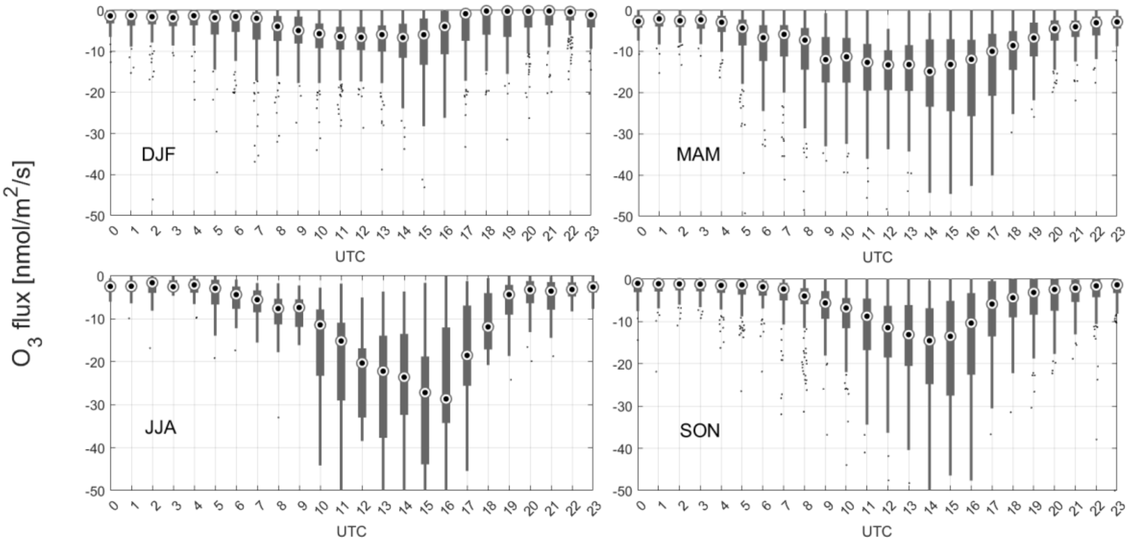

Figure S9: Diurnal variation of ozone fluxes during the seasons (DJF: winter, MAM: spring, JJA, summer, SON: autumn)

Analysis Code:

Dry deposition scheme: Canresistance.m

Flux triad: triad_bessel_NOX.m;

REFERENCES

1. A. J. Haagen-Smit, Chemistry and physiology of Los Angeles smog. *Ind. Eng. Chem.* **44**, 1342–1346 (1952).

2. P. J. Crutzen, The role of NO and NO_2 in the Chemistry of the troposphere and stratosphere. *Annu. Rev. Earth Planet. Sci.* **7**, 443–472 (1979).

3. J. L. Laughner, R. C. Cohen, Direct observation of changing NO_x lifetime in North American cities. *Science.* **366**, 723–727 (2019).

4. Y. Zhang, Y. Wang, G. Chen, C. Smeltzer, J. Crawford, J. Olson, J. Szykman, A. J. Weinheimer, D. J. Knapp, D. D. Montzka, A. Wisthaler, T. Mikoviny, A. Fried, G. Diskin, Large vertical gradient of reactive nitrogen oxides in the boundary layer: Modeling analysis of DISCOVER-AQ 2011 observations. *J. Geophys. Res.* **121**, 1922–1934 (2016).

5. P. O. Wennberg, D. Dabdub, Rethinking ozone production. *Science* **319**, 1624–1625 (2008).

6. B. C. Baier, W. H. Brune, D. O. Miller, D. Blake, R. Long, A. Wisthaler, C. Cantrell, A. Fried, B. Heikes, S. Brown, E. McDuffie, F. Flocke, E. Apel, L. Kaser, A. Weinheimer, Higher measured than modeled ozone production at increased NO_x levels in the Colorado front range. *Atmos. Chem. Phys.* **17**, 11273–11292 (2017).

7. S. C. Anenberg, J. Miller, R. Minjares, L. Du, D. K. Henze, F. Lacey, C. S. Malley, L. Emberson, V. Franco, Z. Klimont, C. Heyes, Impacts and mitigation of excess diesel-related NO_x emissions in 11 major vehicle markets. *Nature* **545**, 467–471 (2017).

8. M. Almaraz, E. Bai, C. Wang, J. Trousdell, S. Conley, I. Faloona, B. Z. Houlton, Agriculture is a major source of NO_x pollution in California. *Sci. Adv.* **4**, eaao3477 (2018).

9. F. Palmgren, R. Berkowicz, O. Hertel, E. Vignati, Effects of reduction of NO_x on the NO_2 levels in urban streets. *Sci. Total Environ.* **189-190**, 409–415 (1996).

10. Y. Fraigneau, M. Gonzalez, A. Coppalle, Turbulence effects upon the NO_2 - NO conversion in the vicinity of an urban area. *Sci. Total Environ.* **189-190**, 293–300 (1996).

11. S. Galmarini, C. Beets, P. G. Duynkerke, J. V.-G. de Arellano, Stable nocturnal boundary layers: A comparison of one-dimensional and large-eddy simulation models. *Boundary-Layer Meteorol.* **88**, 181–210 (1998).

12. M. E. Jenkin, Analysis of sources and partitioning of oxidant in the UK—Part 1: The NO_x-dependence of annual mean concentrations of nitrogen dioxide and ozone. *Atmos. Environ.* **38**, 5117–5129 (2004).

13. R. Velasco, D. Jarosinksa, *WHO Global Air Quality Guidelines* (WHO, 2021).

14. S. K. Grange, A. C. Lewis, S. J. Moller, D. C. Carslaw, Lower vehicular primary emissions of NO_2 in Europe than assumed in policy projections. *Nat. Geosci.* **10**, 914–918 (2017).

15. Z. Jiang, B. C. McDonald, H. Worden, J. R. Worden, K. Miyazaki, Z. Qu, D. K. Henze, D. B. A. Jones, A. F. Arellano, E. V. Fischer, L. Zhu, K. F. Boersma, Unexpected slowdown of US pollutant emission reduction in the past decade. *Proc. Natl. Acad. Sci. U.S.A.* **115**, 5099–5104 (2018).

16. European Environment Agency, "Air quality in Europe – 2015" (Technical Report 5/2015, European Environment Agency, 2015).

17. J. Li, J. Mao, A. M. Fiore, R. C. Cohen, J. D. Crounse, A. P. Teng, P. O. Wennberg, B. H. Lee, F. D. Lopez-Hilfiker, J. A. Thornton, J. Peischl, I. B. Pollack, T. B. Ryerson, P. Veres, J. M. Roberts, J. A. Neuman, J. B. Nowak, G. M. Wolfe, T. F. Hanisco, A. Fried, H. B. Singh, J. Dibb, F. Paulot, L. W. Horowitz, Decadal changes in summertime reactive oxidized nitrogen and surface ozone over the southeast United States. *Atmos. Chem. Phys.* **18**, 2341–2361 (2018).

18. T. Karl, M. Graus, M. Striednig, C. Lamprecht, A. Hammerle, G. Wohlfahrt, A. Held, L. Von Der Heyden, M. J. Deventer, A. Krismer, C. Haun, R. Feichter, J. Lee, Urban eddy covariance measurements reveal significant missing NO_x emissions in Central Europe. *Sci. Rep.* **7**, 2536 (2017).

19. G. Kiesewetter, J. Borken-Kleefeld, W. Schöpp, C. Heyes, P. Thunis, B. Bessagnet, E. Terrenoire, A. Gsella, M. Amann, Modelling NO_2 concentrations at the street level in the GAINS integrated assessment model: Projections under current legislation. *Atmos. Chem. Phys.* **14**, 813–829 (2014).

20. D. A. Burgard, T. R. Dalton, G. A. Bishop, J. R. Starkey, D. H. Stedman, Nitrogen dioxide, sulfur dioxide, and ammonia detector for remote sensing of vehicle emissions. *Rev. Sci. Instrum.* **77**, 014101 (2006).

21. V. Franco, F. P. Sánchez, J. German, P. Mock, Real-world exhaust emissions from modern diesel cars. *ICCT*, 1–52 (2014).

22. D. C. Carslaw, S. Beevers, Estimations of road vehicle primary NO exhaust emission fractions using monitoring data in London. *Atmos. Environ.* **39**, 167–177 (2005).

23. D. H. Lenschow, A. C. Delany, An analytic formulation for NO and NO_2 flux profiles in the atmospheric surface layer. *J. Atmos. Chem.* **5**, 301–309 (1987).

24. P. Leighton, *Photochemistry of Air Pollution* (Academic Press, 1961).

25. J. G. Calvert, W. R. Stockwell, Deviations from the O_3–NO–NO_2 photostationary state in tropospheric chemistry. *Can. J. Chem.* **61**, 983–992 (1983).

26. J. A. Thornton, P. J. Wooldridge, R. C. Cohen, M. Martinez, H. Harder, W. H. Brune, J. Williams, J. M. Roberts, F. C. Fehsenfeld, S. Hall, R. E. Shetter, B. P. Wert, A. Fried, Ozone production rates as a function of NO_x abundances and HO_x production rates in the Nashville urban plume. *J. Geophys. Res.* **107**, ACH 7-1–ACH 7-17 (2002).

27. J. Yang, R. E. Honrath, M. C. Peterson, D. D. Parrish, M. Warshawsky, Photostationary state deviation–Estimated peroxy radicals and their implications for HO_x and ozone photochemistry at a remote northern Atlantic coastal site. *J. Geophys. Res.* **109**, D02312 (2004).

28. B. A. Ridley, S. Madronich, R. B. Chatfield, J. G. Walega, R. E. Shetter, M. A. Carroll, D. D. Montzka, Measurements and model simulations of the photostationary state during the Mauna

Loa observatory photochemistry experiment: Implications for radical concentrations and ozone production and loss rates. *J. Geophys. Res.* **97**, 10375 (1992).

29. L. J. Carpenter, K. C. Clemitshaw, R. A. Burgess, S. A. Penkett, J. N. Cape, G. G. Mcfadyen, Investigation and evaluation of the NO_x/O_3 photochemical steady state. *Atmos. Environ.* **32**, 3353–3365 (1998).

30. R. J. Griffin, P. J. Beckman, R. W. Talbot, B. C. Sive, R. K. Varner, Deviations from ozone photostationary state during the International Consortium for Atmospheric Research on Transport and Transformation 2004 campaign: Use of measurements and photochemical modeling to assess potential causes. *J. Geophys. Res. Atmos.* **112**, D10S07 (2007).

31. K. Mannschreck, S. Gilge, C. Plass-Duelmer, W. Fricke, H. Berresheim, Assessment of the applicability of NO-NO_2 photostationary state to long-term measurements at the Hohenpeissenberg GAW Station, Germany. *Atmos. Chem. Phys.* **4**, 1265–1277 (2004).

32. M. A. K. Khalil, Steady states and transport processes in urban ozone balances. *npj Clim. Atmos. Sci.* **1**, 22 (2018).

33. W. F. Dabberdt, D. H. Lenschow, T. W. Horst, P. R. Zimmerman, S. P. Oncley, A. C. Delany, Atmosphere-surface exchange measurements. *Science* **260**, 1472–1481 (1993).

34. D. H. Lenschow, D. Gurarie, E. G. Patton, Modeling the diurnal cycle of conserved and reactive species in the convective boundary layer using SOMCRUS. *Geosci. Model Dev.* **9**, 979–996 (2016).

35. L. Kristensen, D. H. Lenschow, D. Gurarie, N. O. Jensen, A simple model for the vertical transport of reactive species in the convective atmospheric boundary layer. *Boundary-Layer Meteorol.* **134**, 195–221 (2009).

36. J. Duyzer, G. Deinum, J. Baak, The interpretation of measurements of surface exchange of nitrogen oxides: Correction for chemical reactions. *Philos. Trans. R. Soc. London. Ser. A Phys. Eng. Sci.* **351**, 231–248 (1995).

37. J. V.-G. De Arellano, P. G. Duynkerke, P. J. H. Builtjes, The divergence of the turbulent diffusion flux in the surface layer due to chemical reactions: The NO-O_3-NO_2 system. *Tellus B Chem. Phys. Meteorol.* **45**, 23–33 (2022).

38. Z. Kolbert, J. B. Barroso, R. Brouquisse, F. J. Corpas, K. J. Gupta, C. Lindermayr, G. J. Loake, J. M. Palma, M. Petřivalský, D. Wendehenne, J. T. Hancock, A forty year journey: The generation and roles of NO in plants. *Nitric Oxide* **93**, 53–70 (2019).

39. I. G. Chaparro-Suarez, F. X. Meixner, J. Kesselmeier, Nitrogen dioxide (NO_2) uptake by vegetation controlled by atmospheric concentrations and plant stomatal aperture. *Atmos. Environ.* **45**, 5742–5750 (2011).

40. X. Ren, W. H. Brune, C. A. Cantrell, G. D. Edwards, T. Shirley, A. R. Metcalf, R. L. Lesher, Hydroxyl and peroxy radical chemistry in a rural area of central Pennsylvania: Observations and model comparisons. *J. Atmos. Chem.* **52**, 231–257 (2005).

41. R. Suarez-Bertoa, C. Astorga, V. Franco, Z. Kregar, V. Valverde, M. Clairotte, J. Pavlovic, B. Giechaskiel, *On-Road Vehicle Emissions beyond RDE Conditions* (Ispra, 2019).

42. B. Yang, K. M. Zhang, W. D. Xu, S. Zhang, S. Batterman, R. W. Baldauf, P. Deshmukh, R. Snow, Y. Wu, Q. Zhang, Z. Li, X. Wu, On-road chemical transformation as an important mechanism of NO_2 formation. *Environ. Sci. Technol.* **52**, 4574–4582 (2018).

43. EEA, AirBase; https://eea.europa.eu/data-and-maps/data/airbase-the-european-air-quality-database-8).

44. R. Alvarez, M. Weilenmann, J.-Y. Favez, Evidence of increased mass fraction of NO_2 within real-world NO_x emissions of modern light vehicles derived from a reliable online measuring method. *Atmos. Environ.* **42**, 4699–4707 (2008).

45. Y. Chen, J. Borken-Kleefeld, Real-driving emissions from cars and light commercial vehicles - Results from 13 years remote sensing at Zurich (CH). *Atmos. Environ.* **88**, 157–164 (2014).

46. M. P. Keuken, M. G. M. Roemer, P. Zandveld, R. P. Verbeek, G. J. M. Velders, Trends in primary NO_2 and exhaust PM emissions from road traffic for the period 2000–2020 and implications for air quality and health in the Netherlands. *Atmos. Environ.* **54**, 313–319 (2012).

47. M. Kousoulidou, L. Ntziachristos, G. Mellios, Z. Samaras, Road-transport emission projections to 2020 in European urban environments. *Atmos. Environ.* **42**, 7465–7475 (2008).

48. A. R. Vaughan, J. D. Lee, P. K. Misztal, S. Metzger, M. D. Shaw, A. C. Lewis, R. M. Purvis, D. C. Carslaw, A. H. Goldstein, C. N. Hewitt, B. Davison, S. D. Beevers, T. G. Karl, Spatially resolved flux measurements of NO_X from London suggest significantly higher emissions than predicted by inventories. *Faraday Discuss* **189**, 455–472 (2016).

49. E. J. Dunlea, S. C. Herndon, D. D. Nelson, R. M. Volkamer, F. S. Martini, P. M. Sheehy, M. S. Zahniser, J. H. Shorter, J. C. Wormhoudt, B. K. Lamb, E. J. Allwine, J. S. Gaffney, N. A. Marley, M. Grutter, C. Marquez, S. Blanco, B. Cardenas, A. Retama, C. R. R. Villegas, C. E. Kolb, L. T. Molina, M. J. Molina, Evaluation of nitrogen dioxide chemiluminescence monitors in a polluted urban environment. *Atmos. Chem. Phys.* **7**, 2691–2704 (2007).

50. J. B. A. Muller, C. J. Percival, M. W. Gallagher, D. Fowler, M. Coyle, E. Nemitz, Sources of uncertainty in eddy covariance ozone flux measurements made by dry chemiluminescence fast response analysers. *Atmos. Meas. Tech.* **3**, 163–176 (2010).

51. A. Christen, Atmospheric measurement techniques to quantify greenhouse gas emissions from cities. *Urban Clim.* **10**, 241–260 (2014).

52. T. Foken, B. Wichura, Tools for quality assessment of surface-based flux measurements. *Agric. For. Meteorol.* **78**, 83–105 (1996).

53. X. Lee, W. Massman, B. Law, *Handbook of Micrometeorology* (Kluwer Academic Publishers, 2005); https://springer.com/gp/book/9781402022647.

54. N. Kljun, P. Calanca, M. W. Rotach, H. P. Schmid, A simple two-dimensional parameterisation for flux footprint prediction (FFP). *Geosci. Model Dev.* **8**, 3695–3713 (2015).

55. M. Wesely, Parameterization of surface resistances to gaseous dry deposition in regional-scale numerical models. *Atmos. Environ.* **41**, 52–63 (2007).

56. D. C. Carslaw, S. D. Beevers, G. Fuller, An empirical approach for the prediction of annual mean nitrogen dioxide concentrations in London. *Atmos. Environ.* **35**, 1505–1515 (2001).

57. J. V.-G. de Arellano, A. M. Talmon, P. J. H. Builtjes, A chemically reactive plume model for the NO-NO_2-O_3 system. *Atmos. Environ. Part A. Gen. Top.* **24**, 2237–2246 (1990).

58. M. E. Jenkin, S. M. Saunders, M. J. Pilling, The tropospheric degradation of volatile organic compounds: A protocol for mechanism development. *Atmos. Environ.* **31**, 81–104 (1997).

59. G. M. Wolfe, J. A. Thornton, The chemistry of atmosphere-forest exchange (CAFE) model - Part 1: Model description and characterization. *Atmos. Chem. Phys.* **11**, 77–101 (2011).

60. J. Crawford, D. Davis, J. Olson, G. Chen, S. Liu, G. Gregory, J. Barrick, G. Sachse, S. Sandholm, B. Heikes, H. Singh, D. Blake, Assessment of upper tropospheric HO_x sources over the tropical Pacific based on NASA GTE/PEM data: Net effect on HO_x and other photochemical parameters. *J. Geophys. Res. Atmos.* **104**, 16255–16273 (1999).

61. E. Batchvarova, S.-E. Gryning, Applied model for the growth of the daytime mixed layer. *Boundary-Layer Meteorol.* **56**, 261–274 (1991).

62. D. Leukauf, A. Gohm, M. W. Rotach, Quantifying horizontal and vertical tracer mass fluxes in an idealized valley during daytime. *Atmos. Chem. Phys.* **16**, 13049–13066 (2016).
